# Association between red cell distribution width-to-albumin ratio and prognostic outcomes in pediatric intensive care unit patients: a retrospective cohort study

**DOI:** 10.3389/fped.2024.1352195

**Published:** 2024-03-06

**Authors:** Rui Jing, Baolong Yu, Chenchen Xu, Ying Zhao, Hongmei Cao, Wenhui He, Haili Wang

**Affiliations:** ^1^ Department of Pediatrics, Weifang People’s Hospital, Weifang, Shandong, China; ^2^ Department of Pediatrics, Gaomi Maternal and Child Health Hospital, Weifang, Shandong, China

**Keywords:** critically ill pediatric patients, red cell distribution width-to-Albumin ratio, albumin, pediatric intensive care unit, 28-day mortality rate

## Abstract

**Objective:**

This study aimed to assess the association between Red Cell Distribution Width-to-Albumin Ratio (RAR) and the clinical outcomes in Pediatric Intensive Care Unit (PICU) patients.

**Design:**

This is a retrospective cohort study.

**Methods:**

We conducted a retrospective cohort study based on the Pediatric Intensive Care database. The primary outcome was the 28-day mortality rate. Secondary outcomes included the 90-day mortality rate, in-hospital mortality rate, and length of hospital stay. We explored the relationship between RAR and the prognosis of patients in the PICU using multivariate regression and subgroup analysis.

**Results:**

A total of 7,075 participants were included in this study. The mean age of the participants was 3.4 ± 3.8 years. Kaplan–Meier survival curves demonstrated that patients with a higher RAR had a higher mortality rate. After adjusting for potential confounding factors, we found that for each unit increase in RAR, the 28-day mortality rate increased by 6% (HR = 1.06, 95% CI: 1.01–1.11, *P* = 0.015). The high-RAR group (RAR ≥ 4.0) had a significantly increased 28-day mortality rate compared to the low-RAR group (RAR ≤ 3.36) (HR = 1.7, 95% CI: 1.23–2.37, *P* < 0.001). Similar results were observed for the 90-day and in-hospital mortality rate. No significant interactions were observed in the subgroup analysis.

**Conclusion:**

Our study suggests a significant association between RAR and adverse outcomes in PICU patients. A higher RAR is associated with higher 28-day, 90-day, and in-hospital mortality rates.

## Introduction

Early prognostication of critically ill children is of paramount important as it allows for immediate clinical intervention, thereby improving survival rates. However, due to the inability of children to accurately describe their condition, and their poorer compliance with clinical examinations, the early assessment of critically ill children is more challenging compared to that of adults. Despite the adoption of various critical assessment systems for children in recent years, significant progress in pediatric intensive care and critical care management in China, the overall mortality rate of patients in Chinese pediatric intensive care units (PICUs) remains higher than that in developed countries (1). Moreover, owing to marked variations in medical care levels across different regions of China, the quest for simple and easily obtainable prognostic markers is paramount. This quest is vital for enhancing the ability to identify critically ill children, enabling them to receive more suitable interventions upon admission to the Pediatric Intensive Care Unit (PICU). This is crucial for the early detection and timely treatment of critically ill children.

Red blood cell distribution width (RDW) is a commonly used hematological parameter,which reflects the heterogeneity in the size of red blood cells. Studies suggest that systemic infection and inflammation can inhibit erythropoietin production, affecting red blood cell maturation and increasing the proportion of immature red blood cells in circulation (2). Inflammatory factors can also impact red blood cell membrane glycoproteins and ion channels, leading to changes in red blood cell morphology (3). These pathological changes contribute to increased heterogeneity in red blood cell volume, resulting in elevated RDW. Thus, several studies have suggested that RDW can serve as a novel biomarker for inflammation and oxidative stress (4, 5). Due to its ease of acquisition and cost-effectiveness, RDW has been successfully utilized to prognosticate various diseases, including diabetes and cardiovascular, kidney, and liver diseases (6, 7, 8). Additionally, there is a growing body of evidence supporting the combined use of RDW with other biomarkers to enhance its predictive value for certain diseases (9). Serum albumin, a negative acute-phase protein primarily synthesized by the liver, not only reflects the nutritional status of the body but also exerts anti-inflammatory effects by reducing oxidative stress and inhibiting endothelial cell apoptosis (10, 11). Previous research demonstrates that diminished serum albumin as a risk factor for death in patients with prolonged sepsis (12), and that hospitalized patients with reduced albumin levels have higher mortality rates (13). The same correlation can also be observed in critically ill pediatric patients (14). Nevertheless, albumin levels are susceptible to the influence of nutritional status, inflammation, and chronic illnesses, particularly hepatic dysfunction (15, 16). Several studies have investigated the potential of integrating albumin with other inflammatory markers to enhance its predictive reliability (14, 17).

The RDW-to-Albumin Ratio (RAR) is a new index combining RDW and albumin. It is a novel and simple inflammatory marker that comprehensively reflects hematopoietic dysfunction and hypoalbuminemia. It has garnered widespread attention due to its stability and ease of acquisition. Existing research has demonstrated the effectiveness of RAR in predicting the long-term prognosis of various diseases. Gao C et al. (18) found a significant correlation between RAR and overall mortality and the potential use of renal replacement therapy in severe acute kidney injury (AKI) patients. Lu C et al. (19) observed a significant correlation between RAR and overall mortality in cancer patients. Similar conclusions have been drawn in other diseases such as stroke, acute respiratory distress syndrome (20), and sepsis (9). However, due to RAR being an emerging inflammatory marker, there is currently no consensus on its clinical applications, such as diagnostic scope. Moreover, most existing studies were conducted in adult populations, and there is limited research related to children. Thus, it remains unclear whether RAR is associated with the long-term prognosis of critically ill children.

To identify simple and easily obtainable prognostic markers and enhance the capability to identify critically ill children, we conducted this study to investigate the relationship between RAR and outcomes in PICU patients.

## Materials and methods

### Data source

This study extracted data from the Pediatric Intensive Care (PIC) database, a large, open, specialized, and single-center pediatric database (21). The database comprises clinical data from 12,881 pediatric patients with 13,941 hospitalizations in various ICUs at the Children's Hospital, Zhejiang University School of Medicine, China, from 2010 to 2018. The collected information included basic demographic data, diagnoses, laboratory test results, microbiological results, medication records, clinical outcomes, and vital signs during surgical procedures. The project obtained approval from the Institutional Review Board of the Children's Hospital, Zhejiang University School of Medicine, Hangzhou, China. As the study did not involve any interventions in clinical treatment, explicit consent from patients was not required.

### Study population

**Figure 1:**
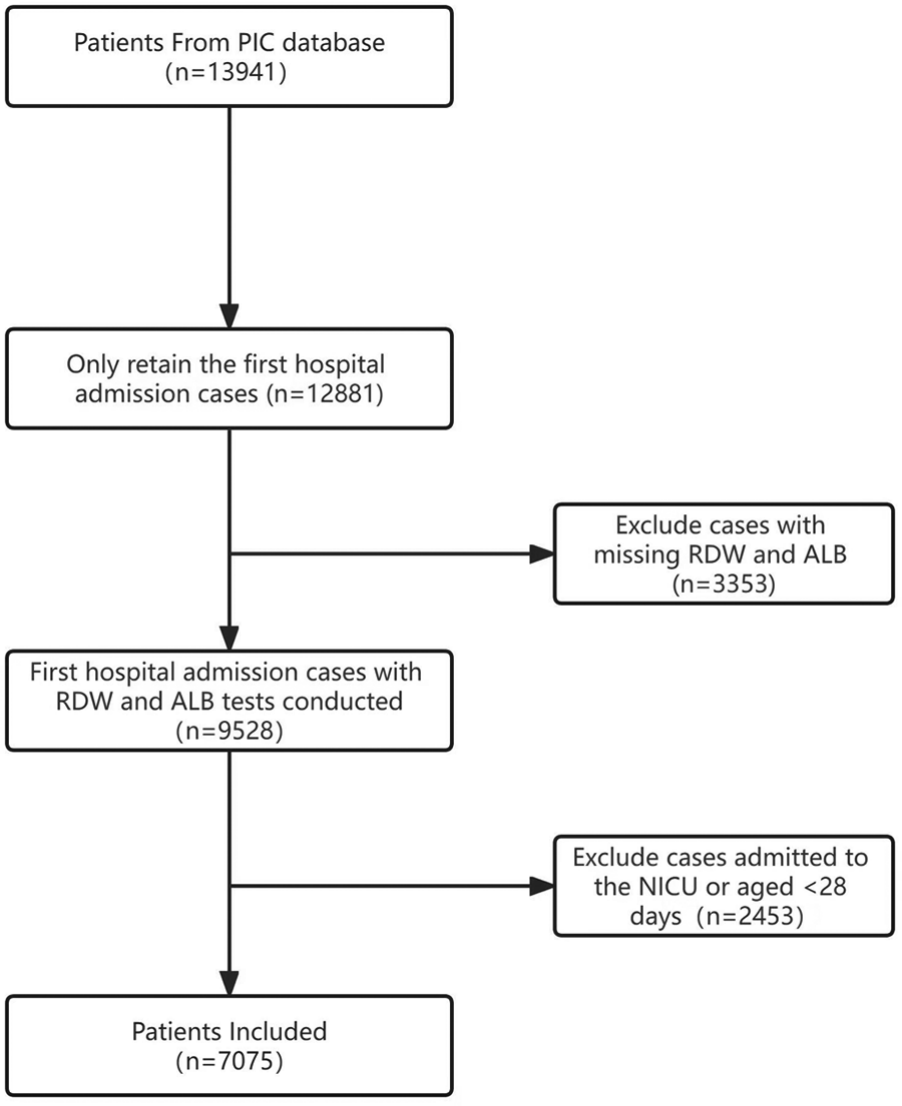
Flowchart of study patient enrollment.

This study was a retrospective cohort study. We included all non-NICU patients aged ≥28 days and <18 years. For children with multiple hospitalizations, only data from the first admission were extracted. Patients lacking data on RDW or albumin were excluded. Additionally, individuals who were missing substantial clinical data were also excluded. Baseline data for the excluded non-neonatal cases are described in Supplementary Table S4. In total, data from 7,075 patients were included in the final analysis. We did not impose any restrictions on the time of in-hospital mortality in this study. Details of the study population are illustrated in Figure 1.

### Data extraction

The extracted data encompassed patient demographics including age, gender, ICU admission type, vital signs (temperature, respiratory rate, heart rate), length of hospital stay, and laboratory test results. The laboratory tests included white blood cell count, neutrophil count, hemoglobin, platelet count, hematocrit, RDW, C-reactive protein, alanine transaminase, aspartate transaminase, total protein, albumin, total bilirubin, direct bilirubin, triglycerides, creatine kinase-MB, lactate dehydrogenase, creatinine, blood urea nitrogen, amylase, anion gap, sodium, glucose, lactate, partial pressure of carbon dioxide, activated partial thromboplastin time, international normalized ratio, D-dimer, fibrinogen, plasma prothrombin time, and thrombin time. Comorbidities, including anemia, hypertension, bacteremia, acute kidney injury, and malignant tumors, were also recorded. The diagnostic criteria for comorbidities are provided in Supplementary Table S1.

The RDW-to-Albumin Ratio (RAR, %/g/dl) was calculated by dividing the RDW by the albumin level. The Los hospital (day) is equal to the time when the child finally leaves the hospital minus the time when the child entered the hospital, measured in days.

All laboratory tests data used were the initial values when patients first entered the ICU, and the comorbidities (anemia, hypertension, acute kidney injury, bacteremia) was calculated from all relevant examination results after admission to the ICU. The primary outcome measure was the mortality rate within 28 days of hospitalization, while the secondary outcomes included the 90-day mortality rate, in-hospital mortality rate, and length of hospital stay.

In cases with more than 40% missing values, covariates were excluded. For continuous variables, virtual variables were used to indicate any missing covariate values. For categorical variables, statistical estimates of missing covariate data were substituted for the missing values.

### Statistical analysis

Study participants were categorized into three groups based on tertiles of RAR for descriptive analysis. Continuous variable data were presented as mean ± standard deviation or median with interquartile range, and differences between groups were compared using *t*-tests or one-way analysis of variance. For categorical variables, data were expressed as frequencies or percentages and analyzed using chi-square tests or Fisher's exact tests.

Multivariate Cox proportional hazards models were constructed to assess the associations between RAR and 28-day and 90-day mortality rates as well as to calculate hazard ratios (HRs). Logistic and linear regression were employed to evaluate the relationships between RAR and in-hospital mortality rate and length of hospital stay, respectively.

In the multivariate regression analysis models, covariates were selected for adjustment based on their correlation with clinical outcomes or when the change in effective estimate exceeded 10%. Model I had no adjusted covariates. Model II was adjusted for gender, age, and ICU type. Model III was additionally adjusted for hypertension, sepsis, acute kidney injury, and malignant tumors. In Model IV, further adjustments were made for white blood cell count, neutrophil count, hemoglobin, platelet count, hematocrit, C-reactive protein, alanine transaminase, gamma-glutamyl transferase, total protein, total bilirubin, direct bilirubin, triglycerides, creatine kinase-MB, lactate dehydrogenase, creatinine, blood urea nitrogen, amylase, anion gap, sodium, glucose, lactate, partial pressure of carbon dioxide, activated partial thromboplastin time, international normalized ratio, D-dimer, fibrinogen, plasma prothrombin time, thrombin time, mechanical ventilator use, and vasopressor use. Smooth curve fitting was employed to assess the relationship between RAR and 28-day mortality in PICU patients. Kaplan–Meier curves were used to compare the survival probabilities of patients with different RAR levels. All analyses were conducted using the R 3.3.2 software package (http://www.R-project.org, R Foundation) and Free Statistics software version 1.8. Two-tailed tests were performed, and a *P*-value < 0.05 was considered statistically significant.

### Subgroup analysis and sensitivity analysis

**Figure 2:**
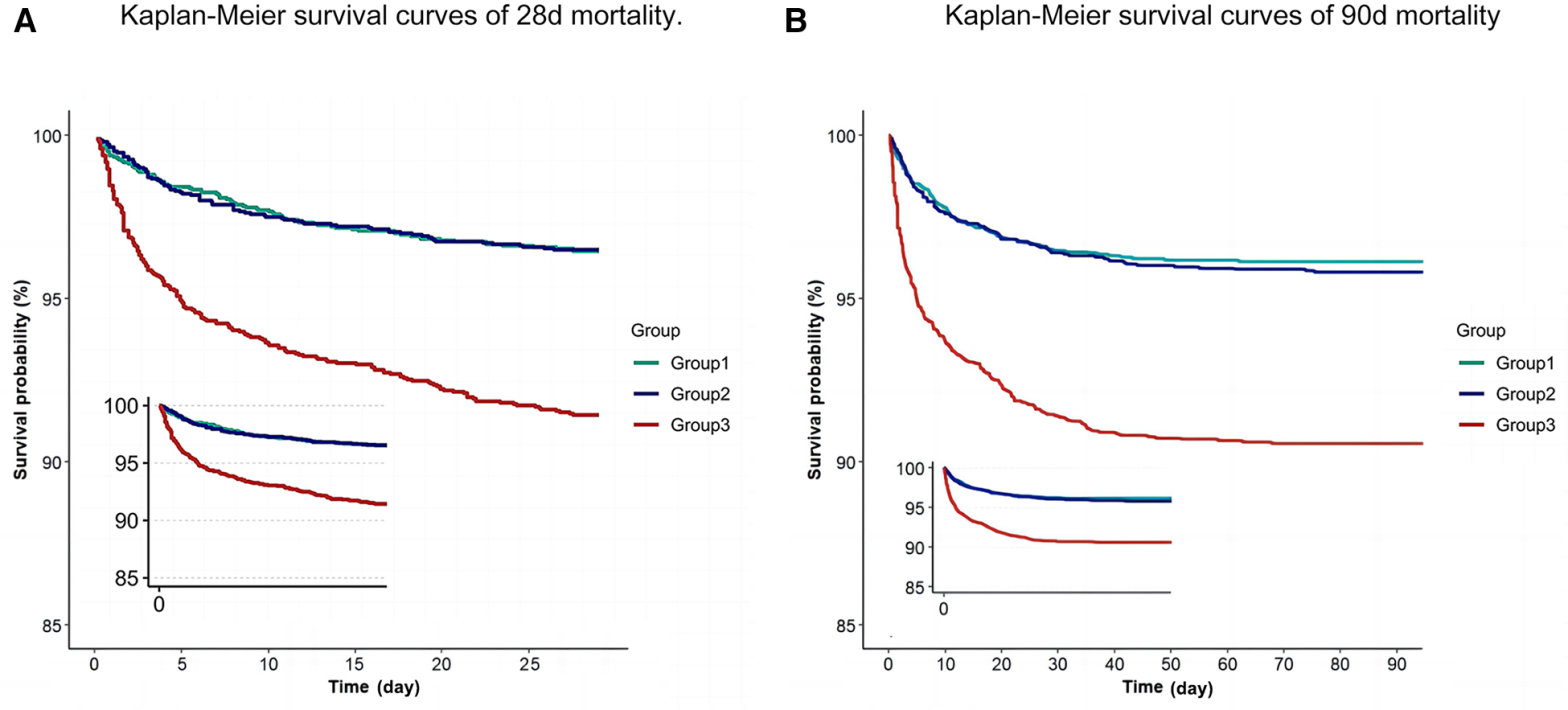
Kaplan–Meier curve of 28-day mortality for patients in PICU (**A**) Kaplan–Meier curve of 90-day mortality for patients in PICU (**B**).

To exclude the potential confounding effects of comorbidity type on the relationship between RAR and 28-day mortality rate, we conducted analyses within the various subgroups. These subgroups included factors such as gender, age, ICU type, comorbidities such as hypertension, bacteremia, acute kidney injury, malignant tumors, and anemia, as well as vasopressor use. The results revealed no significant differences in the correlation between RAR and 28-day mortality rate among these subgroups (Figure 2).

Furthermore, we performed a sensitivity analysis employing both simple and multiple imputation methods to validate missing data. Comparative analysis demonstrated no significant differences in the results between different imputation methods, indicating the robustness of our findings in handling missing data. This sensitivity analysis reinforces our confidence in the study results and ensures the internal consistency of the study.

## Results

### Population and baseline characteristics

From the 13,941 records in the PIC database, 1,060 duplicate cases were excluded. Among the remaining 12,881 hospitalized patients, 5,806 cases were further excluded due to age constraints (<28 days or >18 years) and insufficient data on albumin or RDW. Consequently, a total of 7,075 pediatric cases were included in the analysis (Figure 1).

The demographic details of all study participants are summarized in Table 1. The average age of the patients was 3.4 years, and there were 3,163 females (44.7%) and 3,912 males (55.3%). The mean baseline RAR was 4.0 ± 1.3% /g/dl. Stratification based on RAR values resulted in three groups (<3.36, 3.36–4.02, >4.02). Patients with a higher RAR exhibited increased respiratory and heart rates, RDW, prothrombin time, activated partial thromboplastin time, thrombin time, alanine aminotransferase, gamma-glutamyl transferase, D-dimer, direct bilirubin, triglycerides, and lactate dehydrogenase. Conversely, they demonstrated lower mean arterial pressure, neutrophil count, hemoglobin, hematocrit, platelet count, indirect bilirubin, serum creatinine, amylase, sodium, fibrinogen, anion gap, total protein, albumin, and amylase (Table 2). Notably, the Cardiac ICU had proportionally fewer patients with a high RAR, while the PICU and Surgical ICU exhibited a higher incidence of high-RAR cases. The high-RAR group showed a significantly higher prevalence of malignant tumors and anemia, along with a higher prevalence of bacteremia and vasopressor usage. Furthermore, the high-RAR group had a lower proportion of males and a higher proportion of females.

**Table 1:** Clinical characteristics of the study population.

	Total	Group 1(<3.36)	Group 2 (3.36–4.02)	Group 3(>4.02)	*p*-value
	(*n* = 7,075)	(*n* = 2,356)	(*n* = 2,359)	(*n* = 2,360)	
Sex, *n* (%)					<0.001
Female	3,163 (44.7)	1,148 (48.7)	1,035 (43.9)	980 (41.5)	
Male	3,912 (55.3)	1,208 (51.3)	1,324 (56.1)	1,380 (58.5)	
Age (years)	3.4 ± 3.8	4.5 ± 4.1	2.9 ± 3.5	2.7 ± 3.7	<0.001
Los hospital (day)	14.3 ± 14.5	11.8 ± 10.9	14.3 ± 12.9	16.9 ± 18.3	<0.001
Los ICU (day)	5.4 ± 11.8	5.0 ± 9.6	5.0 ± 10.7	6.1 ± 14.4	0.001
Vital signs					
Temperature (°C)	36.8 ± 0.9	36.8 ± 1.0	36.8 ± 0.8	36.9 ± 0.9	0.451
Breathing rate (beats/min)	29.0 ± 11.2	27.1 ± 7.3	29.0 ± 7.5	31.2 ± 16.4	<0.001
Heart rate (beats/min)	126.7 ± 26.4	119.4 ± 27.7	129.0 ± 25.2	131.6 ± 24.8	<0.001
MAP (mmHg)	77.3 ± 15.7	79.5 ± 16.0	76.6 ± 14.0	75.6 ± 16.8	<0.001
ICU Type *n* (%)					<0.001
CICU	2,145 (30.3)	874 (37.1)	831 (35.2)	440 (18.6)	
General ICU	1,308 (18.5)	490 (20.8)	319 (13.5)	499 (21.1)	
PICU	1,461 (20.7)	462 (19.6)	424 (18)	575 (24.4)	
SICU	2,161 (30.5)	530 (22.5)	785 (33.3)	846 (35.8)	
In-hospital mortality, *n* (%)					<0.001
No	6,658 (94.1)	2,263 (96.1)	2,258 (95.7)	2,137 (90.6)	
Yes	417 (5.9)	93 (3.9)	101 (4.3)	223 (9.4)	
28-day mortality, *n* (%)					<0.001
No	6,710 (94.8)	2,274 (96.5)	2,278 (96.6)	2,158 (91.4)	
Yes	365 (5.2)	82 (3.5)	81 (3.4)	202 (8.6)	
90-day mortality, *n* (%)					<0.001
No	6,662 (94.2)	2,265 (96.1)	2,260 (95.8)	2,137 (90.6)	
Yes	413 (5.8)	91 (3.9)	99 (4.2)	223 (9.4)	
Acute kidney injury					0.004
No	3,819 (55.4)	1,254 (55.1)	1,338 (57.9)	1,227 (53.1)	
Yes	3,073 (44.6)	1,020 (44.9)	971 (42.1)	1,082 (46.9)	
Malignancy, *n* (%)					<0.001
No	6,830 (96.5)	2,313 (98.2)	2,299 (97.5)	2,218 (94)	
Yes	245 (3.5)	43 (1.8)	60 (2.5)	142 (6)	
Anemia, *n* (%)					<0.001
No	3,094 (44.0)	1,195 (50.9)	1,110 (47.3)	789 (33.7)	
Yes	3,941 (56.0)	1,155 (49.1)	1,237 (52.7)	1,549 (66.3)	
Hypertension, *n* (%)					<0.001
No	3,511 (82.6)	1,161 (79.4)	1,265 (84.4)	1,085 (84)	
Yes	740 (17.4)	301 (20.6)	233 (15.6)	206 (16)	
Ventilator use, *n* (%)					0.076
No	6,929 (97.9)	2,306 (97.9)	2,300 (97.5)	2,323 (98.4)	
Yes	146 (2.1)	50 (2.1)	59 (2.5)	37 (1.6)	
Bacteremia, *n* (%)					<0.001
No	5,156 (72.9)	1,870 (79.4)	1,747 (74.1)	1,539 (65.2)	
Yes	1,919 (27.1)	486 (20.6)	612 (25.9)	821 (34.8)	
Vasopressors, *n* (%)					<0.001
No	4,376 (61.9)	1,582 (67.1)	1,398 (59.3)	1,396 (59.2)	
Yes	2,699 (38.1)	774 (32.9)	961 (40.7)	964 (40.8)	

ICU, intensive care unit; CICU, cardiac intensive care unit; PICU, pediatric intensive care unit; SICU, surgical intensive care unit.

**Table 2:** Laboratory information of the study population.

	Total	Group 1 (<3.36)	Group 2 (3.36–4.02)	Group 3 (>4.02)	*p*-value
	(*n* = 7,075)	(*n* = 2,356)	(*n* = 2,359)	(*n* = 2,360)	
WBC(×109)	9.5 (6.5, 13.7)	9.6 (7.0, 13.4)	9.3 (6.4, 13.3)	9.4 (6.0, 14.5)	0.012
NEUT(×109)	6.2 (3.7, 10.0)	6.8 (4.3, 10.3)	6.2 (3.6, 9.7)	5.8 (3.1, 9.8)	<0.001
HGB(g/L)	102.0 (80.0, 118.0)	109.0 (85.0, 124.0)	104.0 (87.0, 117.0)	95.0 (66.0, 110.0)	<0.001
PLT(×109)	260.0 (169.0, 354.0)	266.0 (192.0, 340.0)	263.0 (174.0, 357.0)	249.0 (133.0, 378.0)	<0.001
HCT(%)	32.8 ± 6.9	35.1 ± 6.2	32.8 ± 6.0	30.5 ± 7.7	<0.001
RDW(%)	14.2 ± 2.4	12.8 ± 0.9	13.7 ± 1.1	16.1 ± 3.1	<0.001
CRP (mg/L)	14.0 (4.0, 46.6)	12.0 (4.0, 44.0)	16.0 (4.0, 46.1)	15.0 (4.8, 48.0)	0.003
ALT (U/L)	23.0 (14.0, 37.0)	20.0 (13.0, 31.0)	23.0 (14.0, 35.0)	27.0 (15.0, 59.0)	<0.001
GGT (U/L)	12.0 (9.0, 25.0)	12.0 (9.0, 17.0)	12.0 (9.0, 23.0)	16.0 (10.0, 47.0)	<0.001
TP (g/L)	57.6 ± 9.0	64.0 ± 6.7	57.1 ± 6.4	51.5 ± 8.8	<0.001
ALB (g/L)	37.4 ± 6.0	42.5 ± 3.5	37.6 ± 3.1	32.2 ± 5.6	<0.001
T_BIL (μmol/L)	9.9 (6.2, 16.3)	9.8 (6.3, 14.4)	9.8 (6.3, 15.9)	10.4 (6.0, 20.8)	<0.001
DBIL (μmol/L)	2.6 (1.6, 4.4)	2.4 (1.6, 3.7)	2.5 (1.7, 4.3)	2.9 (1.6, 6.3)	<0.001
TG (mmol/L)	0.8 (0.6, 1.1)	0.7 (0.5, 1.0)	0.8 (0.5, 1.1)	0.9 (0.6, 1.5)	<0.001
CK_MB (U/L)	33.0 (21.0, 54.0)	32.0 (21.0, 51.0)	34.0 (22.0, 54.0)	33.0 (21.0, 58.0)	0.024
LDH (U/L)	370.0 (273.0, 535.0)	334.0 (262.0, 460.0)	371.0 (271.0, 522.0)	416.0 (296.0, 639.0)	<0.001
Anion gap	8.4 (4.7, 12.4)	9.2 (5.7, 13.2)	8.1 (4.5, 11.8)	7.7 (3.9, 11.9)	<0.001
Na (mmol/L)	137.8 ± 5.3	138.5 ± 4.8	138.1 ± 5.0	136.6 ± 5.7	<0.001
GLU (mmol/L)	7.1 (5.8, 9.5)	6.9 (5.7, 9.2)	7.4 (5.9, 9.9)	7.1 (5.7, 9.7)	<0.001
Lac (mmol/L)	1.8 (1.3, 2.6)	1.8 (1.3, 2.6)	1.8 (1.2, 2.5)	1.8 (1.3, 2.8)	0.064
PSO2	37.2 (32.2, 42.9)	36.9 (32.4, 42.2)	37.8 (32.7, 43.2)	36.8 (31.6, 43.2)	0.004
PT (s)	14.4 ± 6.2	13.5 ± 3.8	14.3 ± 6.5	15.4 ± 7.5	<0.001
APTT (s)	32.7 (27.7, 41.5)	30.4 (26.5, 36.5)	32.7 (27.8, 41.0)	35.5 (29.1, 47.5)	<0.001
Fib	2.3 ± 1.0	2.4 ± 0.9	2.3 ± 0.9	2.2 ± 1.1	<0.001
INR	1.2 ± 0.5	1.1 ± 0.3	1.2 ± 0.5	1.3 ± 0.6	<0.001
TT (s)	19.9 ± 6.1	18.9 ± 5.0	19.3 ± 5.0	21.4 ± 7.7	<0.001
D-Di	1.2 (0.7, 2.4)	1.0 (0.5, 1.9)	1.1 (0.6, 2.0)	1.8 (0.9, 3.8)	<0.001
RAR (%/g/dl)	4.0 ± 1.3	3.0 ± 0.2	3.7 ± 0.2	5.2 ± 1.7	<0.001
BUN (mmol/L)	3.3 (2.3, 4.4)	3.5 (2.7, 4.4)	3.0 (2.2, 4.1)	3.2 (2.1, 4.7)	<0.001
SCr (μmol/L)	38.0 (30.0, 46.7)	40.0 (31.0, 50.0)	37.0 (30.0, 44.0)	36.7 (29.0, 46.0)	<0.001
AMY (U/L)	33.4 (16.6, 61.7)	43.3 (26.0, 74.7)	31.0 (15.8, 57.0)	25.1 (13.1, 49.8)	<0.001

Data are weighted estimates, and values are presented as means ± standard deviation or means (percentage).

WBC, white blood cell count; NEUT, neutrophil count; HGB, hemoglobin; PLT, platelet count; HCT, hematocrit; RDW, red blood cell distribution width; CRP, c-reactive protein; ALT, alanine transaminase; GGT, gamma-glutamyl transferase; TP, total protein; ALB, serum albumin; T_BIL, total bilirubin; D_BIL, direct bilirubin; TG, triglycerides; CK-MB, creatine kinase-MB; LDH, lactate dehydrogenas; Na, sodium; GLU, glucose; Lac, lactate; PSO2, partial pressure of carbon dioxide; PT, plasma prothrombin time; APTT, activated partial thromboplastin time; Fib, fibrinogen; INR, international normalized ratio; TT, thrombin time; D-Di, D-dimer; RAR, red cell distribution width-to-albumin ratio; BUN, blood urea nitrogen; SCr, serum creatinine; AMY, amylase.

At study endpoints, the 28-day, 90-day, and overall in-hospital mortality rates were 5.2%, 5.8%, and 5.9%, respectively. The average length of hospital stay was 14.3 days, with a mean ICU stay of 5.4 days. Patients with elevated RAR exhibited significantly increased mortality rates at both 28 and 90 days, along with a notable extension in hospital stay. The data description based on in-hospital outcomes is submitted in Supplementary Table S2.

### Association between RAR and clinical outcomes

We employed a multivariate Cox regression analysis model to explore the relationship between RAR and the prognosis of patients in the PICU. In the unadjusted model, the correlation between RAR and 28-day in-hospital mortality rate was statistically significant (HR 1.14, 95% CI 1.11–1.17, *P* < 0.001). After adjusting for gender, age, ICU type, hypertension, bacteremia, concomitant acute kidney injury and malignancy, as well as the laboratory parameters and treatment interventions in Model IV, a strong correlation between RAR and 28-day in-hospital mortality rate was observed (HR 1.06, 95% CI 1.01–1.11, *P* = 0.015). This suggests that with an increase in RAR values, the 28-day in-hospital mortality rate also increases. The results indicated that RAR remains an independent prognostic factor for in-hospital mortality in critically ill pediatric patients. Similar results were obtained when analyzing 90-day and overall in-hospital mortality rates (Table 3).

**Table 3:** Hazard ratio (HR) [95% confidence intervals (CIs)] for mortality across groups of ratio of red blood cell distribution width (RDW) to albumin (RAR) level.

Variable	Model I		Model II		Model III		Model IV	
	HR(95% CI)	*p*-value	HR(95% CI)	*p*-value	HR(95% CI)	*p*-value	HR(95% CI)	*p*-value
Primary outcomes								
28-day mortality								
RAR	1.14 (1.11–1.17)	<0.001	1.1 (1.07∼1.14)	<0.001	1.1 (1.07–1.13)	<0.001	1.06 (1.01–1.11)	0.015
Tertile								
Group 1 (<3.36)	Ref		Ref		Ref		Ref	
Group 2 (3.36–4.02)	0.99 (0.73, 1.34)	0.93	1.17 (0.86–1.59)	0.33	1.16 (0.85–1.59)	0.336	1.01 (0.73–1.4)	0.957
Group 3 (>4.02)	2.54 (1.96, 3.28)	<0.001	2.26 (1.74–2.93)	<0.001	2.16 (1.66–2.81)	<0.001	1.7 (1.23–2.37)	0.002
*p* for trend		<0.001		<0.001		<0.001		0.001
Secondary outcomes								
90-day mortality								
RAR	1.14 (1.11, 1.16)	<0.001	1.1 (1.07–1.13)	<0.001	1.09 (1.06–1.12)	<0.001	1.06 (1.01–1.11)	0.007
Tertile								
Group 1 (<3.36)	Ref		Ref		Ref		Ref	
Group 2 (3.36–4.02)	1.09 (0.82,1.44)	0.568	1.29 (0.97–1.72)	0.084	1.25 (0.94–1.67)	0.127	1.1 (0.81–1.48)	0.552
Group 3 (>4.02)	2.53 (1.98,3.23)	< 0.001	2.28 (1.78–2.92)	<0.001	2.06 (1.6–2.65)	<0.001	1.75 (1.28–2.38)	<0.001
*p* for trend		< 0.001		<0.001		<0.001		<0.001
In-hospital mortality^a^								
RAR	1.24 (1.17–1.31)	<0.001	1.15 (1.09–1.21)	<0.001	1.13 (1.07–1.19)	<0.001	1.08 (1.01–1.15)	0.028
Tertile								
Group 1 (<3.36)	Ref		Ref		Ref		1(Ref)	
Group 2 (3.36–4.02)	1.09 (0.82–1.45)	0.564	1.31 (0.97–1.76)	0.078	1.26 (0.93–1.7)	0.133	1.19 (0.85–1.66)	0.309
Group 3 (>4.02)	2.54 (1.98–3.26)	<0.001	2.32 (1.79–3.01)	<0.001	2.05 (1.57–2.67)	<0.001	1.84 (1.3–2.61)	0.001
*p* for trend		<0.001		<0.001		<0.001		<0.001
Length of hospital stay^b^	0.88 (0.63, 1.14)	<0.001	0.99 (0.74–1.25)	<0.001	0.6 (0.35–0.84)	<0.001	0.4 (0.11–0.69)	0.007

Model I had no adjusted covariates. Model II adjusted for gender, age, and ICU type. Model III adjusted for model II plus hypertension, sepsis, acute kidney injury, and malignant tumors. Model IV, adjusted for Model III plus white blood cell count, neutrophil count, hemoglobin, platelet count, hematocrit, C-reactive protein, alanine transaminase, gamma-glutamyl transferase, total protein, total bilirubin, direct bilirubin, triglycerides, creatine kinase-MB, lactate dehydrogenase, creatinine, blood urea nitrogen, amylase, anion gap, sodium, glucose, lactate, partial pressure of carbon dioxide, activated partial thromboplastin time, international normalized ratio, D-dimer, fibrinogen, plasma prothrombin time, thrombin time, mechanical ventilator use, and vasopressor use.

^a^ Logistic regression was used to evaluate the association between RAR and in-hospital mortality. The results were expressed as odds ratio (95% CIs).

^b^ Linear regression was used to evaluate the association between RAR and length of stay. The results were expressed as β (95% CIs).

**Figure 3:**
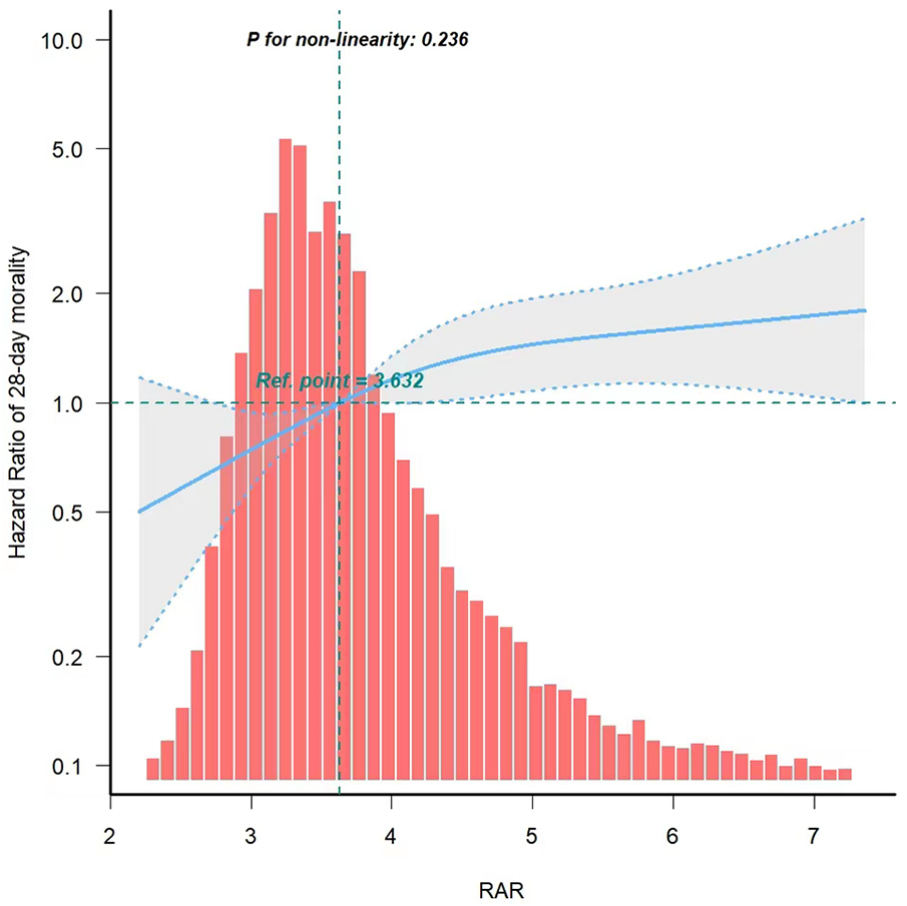
Curve fitting of RAR and 28-day mortality in PICU patients. RAR, Red blood cell distribution width to albumin ratio.

In order to assess the linear correlation between RAR and mortality in PICU patients, we utilized a smooth curve fitting approach. After adjusting for confounding variable, we detected a linear association between RAR and 28-day mortality (Figure 3).

**Figure 4:**
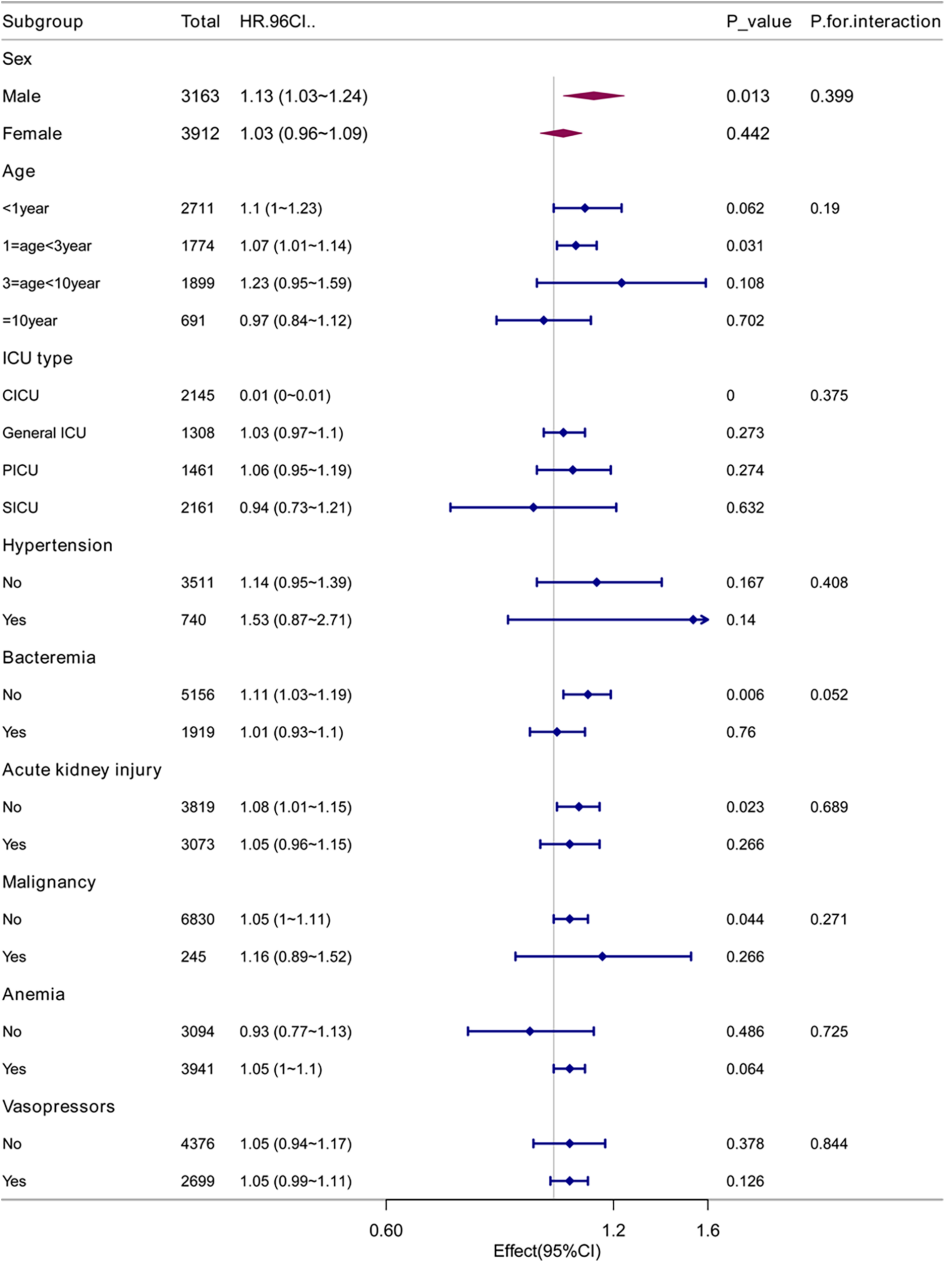
Subgroup analysis of the association between RAR and 28-day mortality in PICU patients.

To evaluate the cumulative survival period at different RAR levels, we stratified patients based on RAR tertiles, generating 28-day survival curves (Figure 4). Kaplan-Meier analysis revealed that the low-RAR group had significantly higher 28-day survival rates compared to the control group (*P* < 0.001). Additionally, similar results were observed in the 90-day survival curves.

Meanwhile, we found that in the KM curve and Cox multivariate analysis, the differences between Group 1 and Group 2 were not significant. Therefore, we conducted a combined analysis of Group 1 and Group 2 and obtained results similar to those described earlier. The results of the combined analysis of Cox multivariate analysis are presented in Supplementary Table S3, and the combined analysis of KM curves is submitted in Supplementary Figure S1.

### Subgroup analysis

We conducted a subgroup analysis using gender, age, ICU type, hypertension, sepsis, acute kidney injury, malignancy, anemia, and vasopressor use as stratification variables to explore the relationship between RAR and 28-day, 90-day, and overall in-hospital mortality rates (Table 4). The analysis revealed no significant interactions between RAR and the various subgroups (P-interaction > 0.05), indicating that the conclusions are stable and reliable across different subgroups (Figure 2).

**Table 4:** Subgroup analysis of the associations between 28-day mortality and the RAR level.

Subgroup	Total	HR(95% CI)	*p*-value	*P* for interaction
Sex				
Male	3,163	1.13 (1.03–1.24)	0.013	0.399
Female	3,912	1.03 (0.96–1.09)	0.442	
Age				
<1 year	2,711	1.1 (1–1.23)	0.062	0.19
1 ≤age <3 year	1,774	1.07 (1.01–1.14)	0.031	
3 ≤age <10 year	1,899	1.23 (0.95–1.59)	0.108	
≥10 year	691	0.97 (0.84–1.12)	0.702	
ICU type				
CICU	2,145	0.01 (0–0.01)	0	0.375
General ICU	1,308	1.03 (0.97–1.1)	0.273	
PICU	1,461	1.06 (0.95–1.19)	0.274	
SICU	2,161	0.94 (0.73–1.21)	0.632	
Hypertension				
No	3,511	1.14 (0.95–1.39)	0.167	0.408
Yes	740	1.53 (0.87–2.71)	0.14	
Bacteremia				
No	5,156	1.11 (1.03–1.19)	0.006	0.052
Yes	1,919	1.01 (0.93–1.1)	0.76	
Acute kidney injury				
No	3,819	1.08 (1.01–1.15)	0.023	0.689
Yes	3,073	1.05 (0.96–1.15)	0.266	
Malignancy				
No	6,830	1.05 (1–1.11)	0.044	0.271
Yes	245	1.16 (0.89–1.52)	0.266	
Anemia				
No	3,094	0.93 (0.77–1.13)	0.486	0.725
Yes	3,941	1.05 (1–1.1)	0.064	
Vasopressors				
No	4,376	1.05 (0.94–1.17)	0.378	0.844
Yes	2,699	1.05 (0.99–1.11)	0.126	

### Receiver operating characteristic analysis

To further evaluate the predictive value of RAR, RDW, albumin, C-reactive protein, and RAR combined with Pediatric Clinical Illness Score (PCIS) for 28-day mortality in critically ill pediatric patients, receiver operating characteristic (ROC) curves were constructed (Supplementary Figure S2). The results showed that the area under the ROC curve (AUC) (95%CI) for RAR, RDW, albumin, C-reactive protein were 0.635 (0.0603, 0.666), 0.609 (0.578, 0.639), 0.596 (0.562, 0.631), and 0.519 (0.490, 0.549), respectively. The AUC (95% CI) of RAR combined with PCIS and PCIS were 0.766 (0.743, 0.788) and 0.747 (0.724, 0.771).

## Discussion

The early identification of critically ill children with poor prognoses is essential for implementing timely clinical interventions and improving survival rates. However, the challenges posed by the diverse and atypical clinical manifestations of pediatric diseases—coupled with children's limited ability to communicate their condition accurately and their lower compliance with clinical examinations—make early assessment more challenging than in adults. Therefore, identifying predictive mortality biomarkers becomes crucial for the early detection and timely treatment of critically ill pediatric patients.

In this study, we found that the RAR serves as a valuable indicator for adverse outcomes in critically ill pediatric patients aged 28 days to 18 years. An increased RAR was significantly associated with an elevated risk of 28-day mortality in patients admitted to the PICU, and similar associations were observed with the 90-day and overall in-hospital mortality rates.

Our findings underscore the importance of RAR as a prognostic biomarker in pediatric critical care. The ability of RAR to predict adverse outcomes provides clinicians with a valuable tool for early risk stratification and intervention, contributing to enhanced management strategies and improved patient outcomes. Its implications as a prognostic indicator warrant further exploration, particularly in the context of its potential integration into routine clinical practice for critically ill pediatric patients.

RDW is a commonly employed hematological parameter, which reflects the heterogeneity in the size of red blood cells. This classic indicator is frequently used in the assessment of circulatory red cell size and size variability, proving valuable in the evaluation of hematologic disorders, infectious diseases, and cardiovascular conditions. The advantages of RDW lie in its ease of acquisition and cost-effectiveness. Studies have demonstrated that systemic infections and inflammation can suppress erythropoietin production, inhibiting red blood cell maturation and increasing the proportion of immature red blood cells circulating in the bloodstream. This phenomenon underscores the sensitivity of RDW as a marker as it reflects alterations in erythropoiesis induced by various pathological conditions. The clinical utility of RDW extends beyond its traditional role, with growing evidence supporting its use as a biomarker for inflammation and oxidative stress in various medical conditions. Its accessibility and affordability make RDW a valuable tool for clinicians in assessing and monitoring patients, particularly in the context of critical care and disease management (2). In addition, inflammatory factors can influence the glycoproteins and ion channels of the red blood cell membrane, altering red blood cell morphology (3, 22). These pathological changes increase the heterogeneity of red blood cell volume, elevating RDW. Consequently, RDW serves as a non-specific marker reflecting inflammation and oxidative stress. RDW has been successfully utilized to prognosticate various diseases, including cardiovascular diseases, kidney diseases, diabetes, tumors, and liver diseases (23–26).

Serum albumin is a crucial protein known for its role in scavenging reactive oxygen species, inhibiting platelet activation and aggregation, and improving blood viscosity (27–29). It plays a significant role in acute inflammatory responses (30). Previous studies have indicated that low serum albumin levels during the acute phase of sepsis serve as a robust predictor of septic shock (31). Further research has demonstrated an increased mortality rate in hospitalized patients with decreased albumin levels (13).

RAR or RDW/ALB is a novel index formed by combining RDW with albumin. It has garnered widespread attention as a comprehensive inflammatory marker appreciated for its stability and ease of acquisition. Studies by Xu et al. (9) identified RAR as a prognostic risk factor for adverse outcomes in critically ill adults with sepsis. Lu Chengdong et al. (19) discovered that elevated RAR is an independent risk factor for increased all-cause mortality in adult cancer patients. Furthermore, RAR has demonstrated good predictive capabilities for the prognosis of patients post-cardiac surgery and adults with acute kidney injury (18, 32). Therefore, RAR may more effectively assess inflammatory responses compared to individual markers like RDW and albumin. However, there is currently a lack of research on the relationship between RAR and the prognosis of critically ill pediatric patients.

In this study, we included 7,075 patients admitted to the PICU. The results indicate an association between RAR and 28-day mortality rate in PICU patients. For each incremental increase in RAR upon ICU admission, there was a 6% rise in the rate of 28-day mortality in pediatric patients (HR 1.06, 95% CI 1.01–1.11, *P* = 0.015). Similar results were observed for the 90-day and in-hospital mortality rates. Additionally, we found that with each one-point increase in RAR upon ICU admission, the length of hospital stay extended by 0.4 days (*β* 0.4, 95% CI 0.11–0.69, *P* = 0.007). This suggests that higher RAR values are associated with poorer clinical prognoses in critically ill pediatric patients.

It is noteworthy that in the high-RAR group, there was a significantly higher proportion of patients with malignancies and anemia. The likelihood of developing sepsis was also higher, and there was a relatively higher trend in vasopressor use. This indicates that RAR not only represents the mathematical combination of RDW and albumin levels but also reflects both hematopoietic dysfunction and hypoalbuminemia. This study further demonstrates that RAR reflects illness severity in PICU patients.

To enhance the reliability of our results, we conducted subgroup analyses based on gender, age, ICU type, hypertension, bacteremia, acute kidney injury, malignancy, anemia, and vasopressor use. The results revealed that a higher RAR was consistently associated with poorer clinical outcomes across all subgroups.

In this study, we observed that the area under the receiver operating characteristic curve for RAR was higher to that for individual measures of RDW or albumin, suggesting that RAR has a higher predictive value for the incidence and mortality of sepsis in pediatric patients over 28 days compared to RDW or albumin alone. We posit that this superiority arises because, in contrast to RDW or albumin alone, RAR not only reflects the patient's hematopoietic and nutritional status but also concurrently mirrors the severity of the patient's illness. Similar conclusions were drawn by Xu et al. (9) in their study of septic adult patients. This suggests that, in the PICU, RAR may better predict illness severity and prognosis relative to RDW or albumin alone. Furthermore, when we combined RAR with the Pediatric Clinical Illness Score (PCIS), the results showed that the combined use of PCIS and RAR improved the predictive ability for critically ill patients and was statistically significant. This suggests that although the independent predictive ability of this indicator is limited compared to various current critical care scores, its predictive ability for critically ill children can be enhanced when used in conjunction with other indicators.

Our study has certain limitations. First, due to the retrospective study design, the population was heterogeneous and comprised children with different diseases. Despite our efforts to adjust for potential confounding factors and perform subgroup analyses, selection and confounding bias are inevitable limitations of all retrospective studies. Second, being a single-center retrospective study, selection bias might influence the accuracy of our results. Therefore, multicenter studies are needed to validate these findings. Third, this study extracted data from the PIC database, which lacked some clinical data points—including baseline features such as weight and systolic blood pressure—for some patients. Considering the potential impact of excessive missing values on the study results, these covariates were not included in the regression analysis. Fourth, this study only extracted the first measured RAR value after ICU admission and did not track its dynamic changes. Fifth, the present study excluded patient cases with missing key RDW and ALB data, and after comparing their baseline data with the included cases, we found that there were certain differences between the two groups of children in terms of gender, length of hospital stay, ICU stay, type of ICU, and in-hospital mortality. Although these differences were not significant, they still had statistical significance and could potentially lead to some statistical bias. Finally, this study primarily provided descriptive results, and causal relationships between RAR values and prognosis were not established. We believe a carefully designed, multicenter prospective study is necessary to validate our findings.

## Conclusion

RAR emerges as a potential prognostic indicator in PICU patients, exhibiting a correlation with adverse clinical outcomes. Elevated RAR levels are associated with increased 28-day, 90-day, and overall in-hospital mortality rates, along with prolonged hospital stays in PICU patients.

## Data Availability

The datasets presented in this study can be found in online repositories. The names of the repository/repositories and accession number(s) can be found in the article/Supplementary Material.
