# Supplementary material for: Association between red cell distribution width-to-albumin ratio and prognostic outcomes in pediatric intensive care unit patients: a retrospective cohort study
Source: Front Pediatr. 2024 Mar 6;12:1352195. doi: 10.3389/fped.2024.1352195 (PMC10950909; doi:10.3389/fped.2024.1352195)
Supplement: Supplementary file 1 [file Datasheet1.pdf]

Supplemental Table

Supplemental Table 1. Diagnostic criteria for complications.

Complications	Diagnostic criteria
**Acute kidney injury**	We adopted the 2018 pROCK standard. AKI is defined as an increase in serum creatinine by≥20μmol/L within 7 days, reaching 1.3 times or more of the baseline value.
**Anemia**	1-4 months < 90 g/L;<br>4-6 months < 100 g/L;<br>6-60minths < 110 g/L;<br>60-144months < 115 g/L;<br>> 144 months < 120 g/L.
**Hypertension**	Systolic pressure > 120 mmHg;<br>diastolic pressure > 80 mmHg.

Supplemental Table 2. Clinical characteristics of the study population based on in-hospital outcomes

	Total	In-hospital mortality		p
	(n = 7075)	No(n = 6658)	Yes(n = 417)	
**Sex**				0.001
female	3163 (44.7)	3008 (45.2)	155 (37.2)	
male	3912 (55.3)	3650 (54.8)	262 (62.8)	
**age(years)**	3.4 ± 3.8	3.4 ± 3.8	3.2 ± 3.9	0.354
**Los hospital (day)**	14.3 ± 14.5	14.4 ± 14.4	12.6 ± 17.1	0.011
**Los ICU (day)**	5.4 ± 11.8	5.0 ± 11.3	11.7 ± 15.7	< 0.001
**Vital sign**				
Temperature (◦C)	36.8 ± 0.9	36.9 ± 0.9	36.7 ± 1.7	0.004
Breathing rate(beats/min)	29.0 ± 11.2	28.8 ± 9.7	37.8 ± 32.2	< 0.001
Heart rate(beats/min)	126.7 ± 26.4	126.5 ± 26.2	134.3 ± 32.7	< 0.001
MAP(mmHg)	77.3 ± 15.7	77.4 ± 15.6	71.2 ± 17.2	< 0.001
**ICU Type n(%)**				< 0.001
CICU	2145 (30.3)	2123 (31.9)	22 (5.3)	
General ICU	1308 (18.5)	1102 (16.6)	206 (49.4)	
PICU	1461 (20.7)	1315 (19.8)	146 (35)	
SICU	2161 (30.5)	2118 (31.8)	43 (10.3)	
**Acute kidney injur, n (%)**				0.002
No	3819 (55.4)	3621 (55.9)	198 (48.1)	
Yes	3073 (44.6)	2859 (44.1)	214 (51.9)	
**Malignancy, n (%)**				< 0.001
No	6830 (96.5)	6451 (96.9)	379 (90.9)	
Yes	245 (3.5)	207 (3.1)	38 (9.1)	
**Anemia, n (%)**				< 0.001
No	3094 (44.0)	3003 (45.3)	91 (22.2)	
Yes	3941 (56.0)	3623 (54.7)	318 (77.8)	
**Hypertension, n (%)**				0.434
No	3511 (82.6)	3428 (82.5)	83 (85.6)	
Yes	740 (17.4)	726 (17.5)	14 (14.4)	
**Ventilator use, n (%)**				0.002
No	6929 (97.9)	6512 (97.8)	417 (100)	
Yes	146 (2.1)	146 (2.2)	0 (0)	
**Bacteremia, n (%)**				< 0.001
No	5156 (72.9)	4920 (73.9)	236 (56.6)	
Yes	1919 (27.1)	1738 (26.1)	181 (43.4)	
**Vasopressors, n (%)**				< 0.001
No	4376 (61.9)	4170 (62.6)	206 (49.4)	
Yes	2699 (38.1)	2488 (37.4)	211 (50.6)	

ICU intensive care unit, **CICU** cardiac intensive care unit,**PICU** pediatric intensive care unit, **SICU** surgical intensive care unit

Supplemental Table 2 (continue). Laboratory information of the study population based on in-hospital outcomes

	Total	In-hospital mortality		p
	(n = 7075)	No(n = 6658)	Yes(n = 417)	
WBC($\times 10^9$)	9.5 (6.5, 13.7)	9.4 (6.5, 13.6)	10.5 (6.2, 16.4)	0.017
NEUT($\times 10^9$)	6.2 (3.7, 10.0)	6.2 (3.7, 10.0)	6.3 (2.8, 10.6)	0.145
HGB(g/L)	102.0 (80.0, 118.0)	103.0 (84.0, 119.0)	12.4 (9.7, 102.0)	< 0.001
PLT($\times 10^9$)	260.0 (169.0, 354.0)	262.0 (173.0, 355.0)	216.0 (106.0, 346.0)	< 0.001
HCT(%)	32.8 ± 6.9	32.9 ± 6.8	31.8 ± 8.8	0.003
RDW(%)	14.2 ± 2.4	14.2 ± 2.4	14.8 ± 2.4	< 0.001
CRP (mg/L)	14.0 (4.0, 46.6)	14.5 (4.0, 46.8)	10.0 (4.0, 46.0)	0.959
ALT(U/L)	23.0 (14.0, 37.0)	23.0 (14.0, 35.0)	37.5 (18.0, 106.0)	< 0.001
GGT(U/L)	12.0 (9.0, 25.0)	12.0 (9.0, 23.0)	27.0 (14.0, 61.0)	< 0.001
TP(g/L)	57.6 ± 9.0	57.7 ± 8.8	54.8 ± 11.7	< 0.001
ALB(g/L)	37.4 ± 6.0	37.5 ± 5.8	35.2 ± 8.0	< 0.001
T_BIL(μmol/L)	9.9 (6.2, 16.3)	10.0 (6.3, 16.2)	8.2 (4.6, 16.7)	< 0.001
DBIL(μmol/L)	2.6 (1.6, 4.4)	2.5 (1.6, 4.3)	3.1 (1.8, 7.2)	< 0.001
TG(mmol/l)	0.8 (0.6, 1.1)	0.8 (0.5, 1.1)	1.1 (0.7, 1.8)	< 0.001
CK_MB(U/L)	33.0 (21.0, 54.0)	33.0 (21.0, 53.0)	39.0 (21.0, 105.0)	< 0.001
LDH(U/L)	370.0 (273.0, 535.0)	365.0 (270.8, 520.0)	543.0 (340.0, 1076.0)	< 0.001

Data are weighted estimates, and values are presented as means ± standard deviation or means (percentage).
WBC white blood cell count,**NEUT** neutrophil count, **HGB** hemoglobin,**PLT** platelet count, **HCT** hematocrit, **RDW** red blood cell distribution width,**CRP** c-reactive protein, **ALT** alanine transaminase, **GGT** gamma-glutamyl transferase,**TP** total protein, **ALB** serum albumin,**T_BIL** total bilirubin, **D_BIL** direct bilirubin, **TG** triglycerides, **CK-MB** creatine kinase-MB, **LDH** lactate dehydrogenas

Supplemental Table 2 (continue). Laboratory information of the study population based on in-hospital outcomes

	Total	In-hospital mortality		P
	(n = 7075)	No(n = 6658)	Yes(n = 417)	
Anion gap	8.4 (4.7, 12.4)	8.2 (4.5, 11.9)	14.3 (10.1, 18.5)	< 0.001
Na(mmol/l)	137.8 ± 5.3	137.8 ± 5.1	137.3 ± 7.1	0.057
GLU(mmol/l)	7.1 (5.8, 9.5)	7.2 (5.8, 9.5)	6.5 (5.2, 10.9)	0.025
Lac(mmol/l)	1.8 (1.3, 2.6)	1.7 (1.2, 2.6)	2.9 (1.7, 6.5)	< 0.001
PSO2	37.2 (32.2, 42.9)	37.1 (32.3, 42.7)	38.0 (30.7, 48.0)	0.102
PT(s)	14.4 ± 6.2	14.2 ± 5.6	18.3 ± 11.9	< 0.001
APTT(s)	32.7 (27.7, 41.5)	32.6 (27.7, 40.9)	38.0 (29.0, 52.5)	< 0.001
Fib	2.3 ± 1.0	2.3 ± 1.0	1.9 ± 1.2	< 0.001
INR	1.2 ± 0.5	1.2 ± 0.5	1.5 ± 1.0	< 0.001
TT(s)	19.9 ± 6.1	19.7 ± 5.8	23.4 ± 9.9	< 0.001
D-Di	1.2 (0.7, 2.4)	1.2 (0.7, 2.3)	3.4 (1.0, 6.7)	< 0.001
RAR (%/g/dL)	4.0 ± 1.3	3.9 ± 1.3	4.6 ± 2.1	< 0.001
BUN(mmol/l)	3.3 (2.3, 4.4)	3.2 (2.3, 4.3)	4.7 (3.2, 6.9)	< 0.001
SCr(μmol/L)	38.0 (30.0, 46.7)	37.0 (30.0, 46.0)	46.0 (33.0, 64.6)	< 0.001
AMY(U/L)	33.4 (16.6, 61.7)	33.4 (16.7, 61.2)	31.7 (14.7, 69.0)	0.935

Na sodium, **GLU** glucose, **Lac** lactate, **PSO2** partial pressure of carbon dioxide, **PT** plasma prothrombin time, **APTT** activated partial thromboplastin time, **Fib** fibrinogen, **INR** international normalized ratio, **TT** thrombin time, **D-Di** D-dimer,**RAR** red cell distribution width-to-albumin ratio, **BUN** blood urea nitrogen, **SCr** serum creatinine, **AMY** amylase

Supplemental Table 3. Hazard ratio (HR) [95% confidence intervals (CIs)] for mortality across groups of ratio of red blood cell distribution width (RDW) to albumin (RAR) level.

	Model Ⅰ		Model Ⅱ		Model Ⅲ		Model Ⅳ	
	HR(95% CI)	p-value	HR(95% CI)	p-value	HR(95% CI)	p-value	HR(95% CI)	p-value
**Primary outcomes**								
28-day mortality								
Group1(＜4.02)	1(Ref)		1(Ref)		1(Ref)		1(Ref)	
Group2(＞4.02)	2.56 (2.08~3.15)	<0.001	2.1 (1.71~2.59)	<0.001	2.01 (1.63~2.49)	<0.001	1.7 (1.31~2.2)	<0.001
**Secondary outcomes**								
90-day mortality								
Group1(＜4.02)	1(Ref)		1(Ref)		1(Ref)		1(Ref)	
Group2(＞4.02)	2.43 (2.01~2.95)	<0.001	2.02 (1.66~2.45)	<0.001	1.84 (1.51~2.25)	<0.001	1.65 (1.3~2.11)	<0.001
In-hospital mortality								
Group1(＜4.02)	1(Ref)		1(Ref)		1(Ref)		1(Ref)	
Group2(＞4.02)	2.38 (1.97~2.89)	<0.001	1.97 (1.62~2.39)	<0.001	1.79 (1.47~2.18)	<0.001	1.61 (1.27~2.05)	<0.001

Model I had no adjusted covariates. **Model II** adjusted for gender, age, and ICU type. **Model III** adjusted for model II plus hypertension, sepsis, acute kidney injury, and malignant tumors. **Model IV**, adjusted for Model III plus white blood cell count, neutrophil count, hemoglobin, platelet count, hematocrit, C-reactive protein, alanine transaminase, gamma-glutamyl transferase, total protein, total bilirubin, direct bilirubin, triglycerides, creatine kinase-MB, lactate dehydrogenase, creatinine, blood urea nitrogen, amylase, anion gap, sodium, glucose, lactate, partial pressure of carbon dioxide, activated partial thromboplastin time, international normalized ratio, D-dimer, fibrinogen, plasma prothrombin time, thrombin time, mechanical ventilator use, and vasopressor use.

Supplemental Table 4.Baseline data for the excluded non-neonatal cases

	Total (n = 9505)	versus included cases (n = 7075)	not included cases (n = 2430)	p
**Sex, n (%)**				0.008
Female	4174 (43.9)	3163 (44.7)	1011 (41.6)	
Male	5331 (56.1)	3912 (55.3)	1419 (58.4)	
**age, Mean ± SD**	3.4 ± 3.9	3.4 ± 3.8	3.5 ± 3.9	0.244
**Los hospital (day)**	13.9 ± 14.2	14.3 ± 14.5	12.8 ± 13.1	< 0.001
**Los ICU (day)**	5.1 ± 11.3	5.4 ± 11.8	4.5 ± 9.9	< 0.001
**Vital signs**				
Temperature (◦C)	36.8 ± 0.9	36.8 ± 0.9	36.7 ± 0.8	< 0.001
Breathing rate(beats/min)	29.1 ± 11.9	29.0 ± 11.2	29.4 ± 14.9	0.367
Heart rate(beats/min)	126.5 ± 26.8	126.7 ± 26.4	125.1 ± 28.4	0.088
MAP(mmHg)	77.4 ± 15.4	77.3 ± 15.7	77.8 ± 13.9	0.338
**ICU Type n(%)**				< 0.001
CICU	2446 (25.7)	2145 (30.3)	301 (12.4)	
General ICU	2616 (27.5)	1308 (18.5)	1308 (53.8)	
PICU	1942 (20.4)	1461 (20.7)	481 (19.8)	
SICU	2501 (26.3)	2161 (30.5)	340 (14)	
**In-hospital mortality, n(%)**				< 0.001
No	8824 (92.8)	6658 (94.1)	2166 (89.1)	
Yes	681 (7.2)	417 (5.9)	264 (10.9)	
**Vasopressors, n (%)**				0.234
No	5912 (62.2)	4376 (61.9)	1536 (63.2)	
Yse	3593 (37.8)	2699 (38.1)	894 (36.8)	

ICU intensive care unit, **CICU** cardiac intensive care unit,**PICU** pediatric intensive care unit, **SICU** surgical intensive care uni

Supplemental Figure

Supplemental Figure 1.Kaplan–Meier curve of 28-day mortality for patients in PICU (A). Kaplan–Meier curve of 90-day mortality for patients in PICU(B).

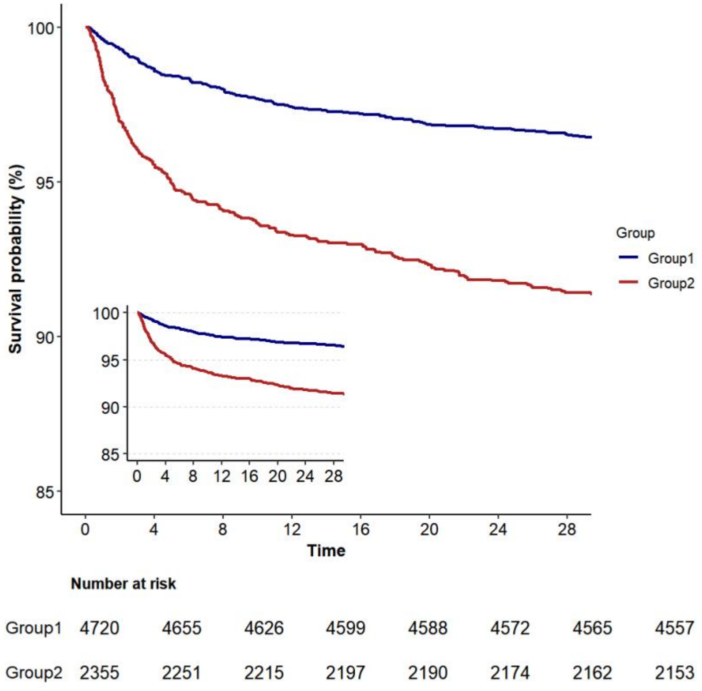

(A) Kaplan–Meier curve of 28-day mortality for patients in PICU

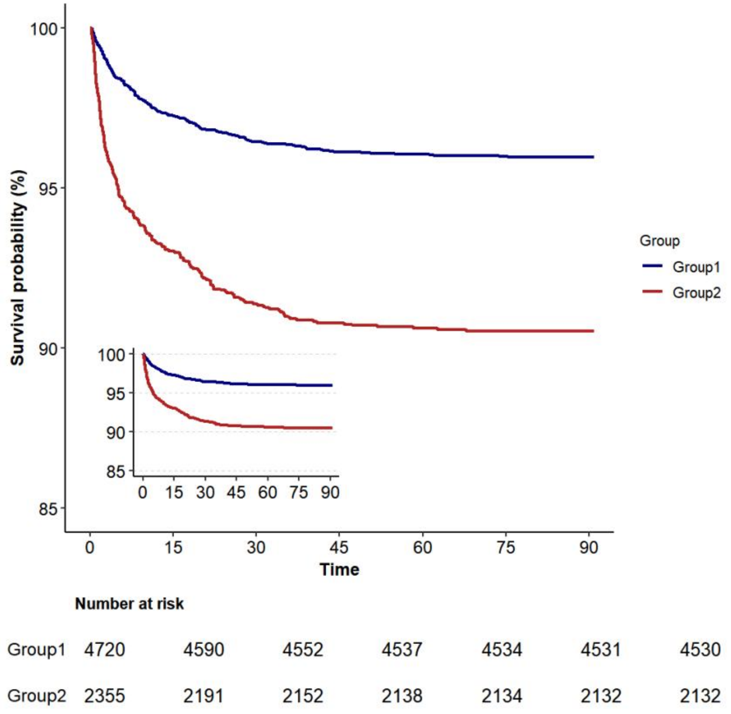

(B) Kaplan–Meier curve of 90-day mortality for patients in PICU

Supplemental Figure 2.Receiver operating characteristic (ROC) curves of the RDW, albumin,C-reactive protein, and RAR combined with Pediatric Clinical Illness Score (PCIS)

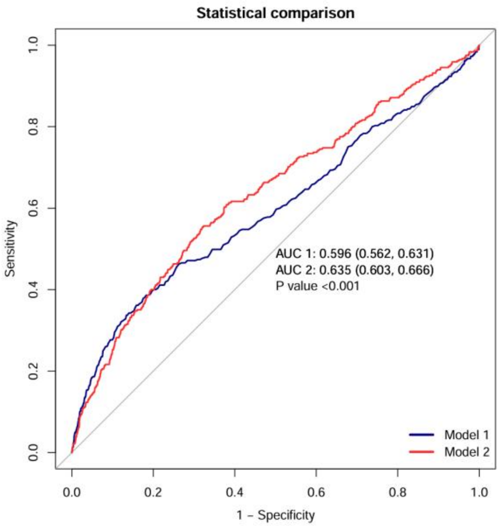

(A) Receiver operating characteristic curves of the RAR (Model 2) and albumin (Model 1)

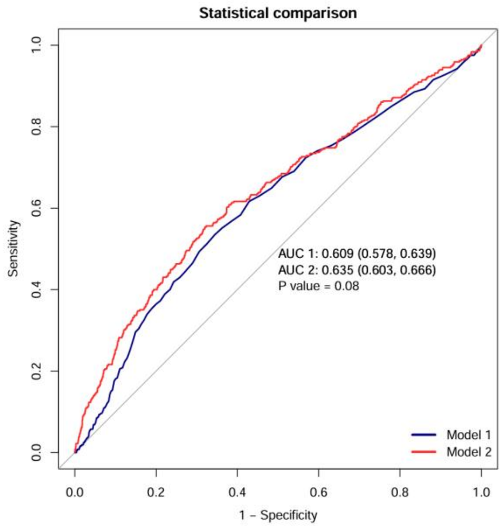

(B) Receiver operating characteristic curves of the RAR (Model 2) and RDW (Model 1)

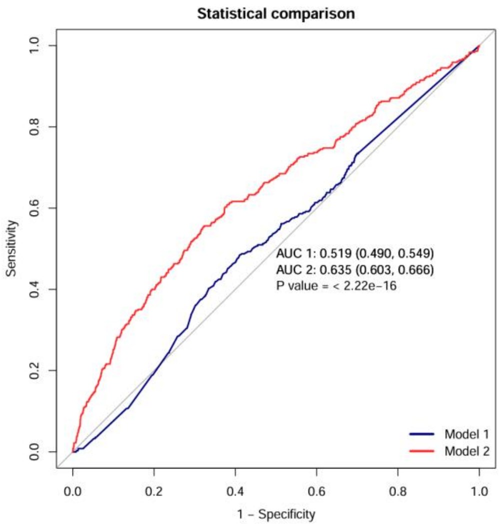

(C) Receiver operating characteristic curves of the RDW (Model 2) and C-reactive protein (Model 1)

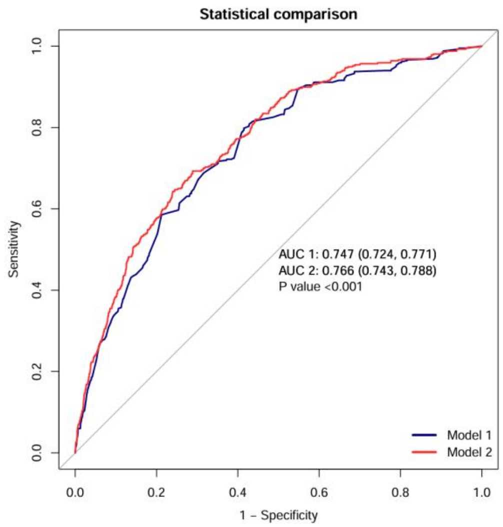

(D) Receiver operating characteristic curves of the PCIS (Model 1) and PCIS+RAR (Model 2)
